# Vascular dysfunction in COPD with and without chronic respiratory failure: a cross-sectional study

**DOI:** 10.3389/fphys.2025.1711419

**Published:** 2026-01-13

**Authors:** Mara Paneroni, Carla Simonelli, Beatrice Salvi, Laura Bertacchini, Michele Vitacca, Massimo Venturelli

**Affiliations:** 1 Respiratory Rehabilitation Unit, Istituti Clinici Scientifici Maugeri IRCCS, Institute of Lumezzane, Brescia, Italy; 2 Department of Neuroscience, Biomedicine and Movement Sciences, University of Verona, Verona, Italy; 3 Department of Internal Medicine, University of Utah, Salt Lake City, UT, United States

**Keywords:** COPD, endothelial dysfunction, nitric oxide, pulmonary rehabilitation, single leg passive movement

## Abstract

**Background:**

Vascular dysfunction has been described as worsening in Chronic Obstructive Pulmonary Disease (COPD), but there is a lack of knowledge regarding severe patients. This retrospective cross-sectional study aimed to investigate it in COPD with Chronic Respiratory Failure (CRF) versus COPD and controls.

**Methods:**

A baseline screening was performed, including a health history, a physical examination, and an anthropometric assessment. All subjects underwent an arterial blood gas analysis, spirometry, a 6-min walking test, and a thigh muscle volume assessment. The vascular function was determined via single Passive Leg Movement (sPLM) on the dominant leg.

**Results:**

Fifteen patients with moderate COPD (FEV_1_ 53.3% ± 11.4%), 15 patients with severe COPD (CRF; FEV_1_ 30.6% ± 10.3%), and 15 age-matched healthy controls (CTRL) were recruited. Reactive hyperemia following sPLM was decreased in CRF [peak LBF (Leg Blood Flow) 70 ± 38 mL/min)] compared to COPD (peak LBF 162 ± 115 mL/min) and CTRL (peak LBF 268 ± 134 mL/min), p < 0.05. Interestingly, when the vascular function was normalized to the thigh muscle volume, the difference in the hyperaemic response among the CRF, COPD, and CTRL groups was mitigated but not eliminated. Moreover, the peak LBF was associated with the 6-min walking test (r = 0.7027, p < 0,0001), FEV_1_ (r = 0.5432, p = 0.0001), disease duration (r = 0.5062, p = 0.0004), oxygen saturation (SpO_2_) (r = 0.4343, p = 0.0029), and prescribed oxygen flow (r = −0.5413, p < 0.0001).

**Conclusion:**

These data provide evidence of an intrinsic vascular dysfunction during the progression of COPD, which depends only partially on locomotor muscle volume loss observed in this clinical population.

## Introduction

Chronic obstructive pulmonary disease (COPD) is a chronic condition that primarily affects the respiratory system (Agustí et al., 2023). To date, many multisystemic consequences of COPD scenario have been widely described, in particular: impaired fitness condition (decreased maximal oxygen uptake), peripheral muscle dysfunction (Maltais et al., 2014), and vascular impairments (Theodorakopoulou et al., 2021; Vaes et al., 2018), which ultimately affect the physical status and quality of life of patients and increase mortality (Screm et al., 2024). Vascular dysfunction refers to the inability of blood vessels to maintain normal function, often due to endothelial dysfunction, impaired vasodilation, and increased vascular tone. Vascular dysfunction plays a central role in the pathophysiology of several cardiovascular and respiratory diseases, contributing to altered blood flow and increased risk of complications (Förstermann, 2010). In fact, a recent meta-analysis of 17 studies (Vaes et al., 2017) showed a significant reduction in vascular function, as measured by flow-mediated dilation (FMD), in patients with COPD. An alternative method for assessing vascular function assessment is Passive Leg Movement (PLM) or single Passive Leg Movement (sPLM) (Broxterman et al., 2017; Venturelli et al., 2017). PLM and FMD are both Doppler ultrasound-based methods that evaluate vasodilatory responses and are sensitive to nitric oxide (NO)-mediated endothelial function. They are all used to detect age- and disease-related vascular dysfunction and have demonstrated construct validity in healthy and diseased populations (Broxterman et al., 2017; Rossman et al., 2016; D'Agata et al., 2022; Hydren et al., 2019).

However, sPLM primarily assesses microvascular (resistance artery) function in the lower limb. This response is predominantly NO-dependent (65%–80%) and minimally affected by metabolic factors. This makes sPLM a direct index of NO bioavailability. In contrast, FMD measures macrovascular (conduit artery) function in the upper limb, reflecting NO-dependent and NO-independent mechanisms (Broxterman et al., 2017; Rossman et al., 2016; D'Agata et al., 2022; Hydren et al., 2019).

In the COPD scenario, Ives et al. (2020) investigated vascular function via PLM in 13 patients with moderate COPD (mean FEV_1_ 53% ± 4% of predicted) and age-matched healthy controls, and reported a significant reduction of PLM-induced hyperemia in the COPD group (Ives et al., 2020), suggesting intrinsic vascular worsening in this population, however it is not clear if other factors associated with the COPD may play a role. The literature also highlights that vascular dysfunction and airflow obstruction follow the same pathway of deterioration during disease progression of pulmonary impairment (Clarenbach et al., 2013; Kingwell, 2000; Piccari et al., 2020). Unfortunately, previous studies have mainly focused on the moderate stages of COPD and have not assessed vascular function in patients with severe COPD and chronic respiratory failure (CRF). In COPD, CRF is defined as the respiratory system’s sustained inability to maintain normal gas exchange. This results in persistent hypoxemia (PaO_2_ < 55–60 mmHg) and/or chronic hypercapnia (PaCO_2_ > 45 mmHg) in a stable clinical state, outside of acute exacerbations. From a pathophysiological point of view, this reflects a progressive decline in ventilatory capacity and gas exchange caused by irreversible airflow limitation, hyperinflation, and abnormal pulmonary mechanics (Budweiser et al., 2008).

CRF is a worsening condition of COPD that manifests itself in the advanced stages of the disease. It is characterized by a severe effort tolerance, a high risk of COPD exacerbations, diminished quality of life, a bad prognosis, and significant healthcare costs (Vitacca et al., 2011). Considering the lack of specific vascular literature in this population, it is imperative to understand if vascular function is further impaired in severe stages of COPD, and from a physiological point of view, it is important to identify potential mechanisms of this pathophysiological phenomenon. Indeed, a constellation of comorbidities is associated with the worsening of the pulmonary and vascular dysfunction as exhibited by the patients with COPD and CRF (Vogelmeier et al., 2025). However, two main factors should be accounted for the correct interpretation of the vascular function assessments in COPD and CRF: patients with COPD typically exhibit severe muscle volume/mass loss of the locomotor limbs (Benz et al., 2019), and this severe muscle wasting must be accounted for, because PLM-induced hyperemia is largely dependent on the muscle volume. In this regard, we have demonstrated that when the difference in blood flow response due to the PLM is normalized by the muscle volume, even in subjects with severe muscle disuse (spinal cord injury), the vascular function is preserved (Venturelli et al., 2014). Similarly, the PLM-induced hyperemia of patients with COPD and CRF must be normalized for the locomotor muscle volume. The second main physiological factor that needs to be accounted, is the interaction with the oxygen blood saturation (SpO_2_) (and potentially O_2_ delivery) with the PLM-induced hyperemia. Wan et al. (2022) demonstrated that acute hypoxia can modulate the hyperaemic response to PLM, likely due to β-adrenergic activation facilitating the peripheral haemodynamic response to PLM.

However, patients with COPD and CRF are usually treated with long-term oxygen therapy when hypoxemia occurs. To date, it is unclear to what extent this therapy may have a protective effect on the development of endothelial damage, especially considering the documented harmful effects of oxygen overuse (Carpagnano et al., 2004).

The first aim of this study was therefore to determine whether vascular function is impaired to a greater extent in patients with CRF than in COPD patients without CRF and in healthy controls. The second aim was to investigate potential contributors to this vascular phenomenon, with specific emphasis on the interactions between locomotor muscle volume, oxygen saturation, and PLM-induced hyperemia. We hypothesized that vascular function in patients with CRF would be further compromised compared to that of COPD patients without CRF. Furthermore, this further vascular impairment might be associated with locomotor muscle wasting and oxygen blood content.

## Methods

### Subjects

We included three different groups of subjects with the following characteristics:

- COPD GROUP: patients affected by moderate COPD at GOLD stage 2 or 3;
- CRF GROUP: patients affected by severe COPD at GOLD stage 4 with CRF;
- CTRL GROUP: healthy volunteers, sex- and age-matched.

The data of COPD GROUP and CRF GROUP refer to two prospective studies conducted at ICS Maugeri, Lumezzane (BS) (funded by the Italian Italian Ministry of Health) between August 2019 and June 2023. The Ethics Committee approved the former study in COPD patients (19 June 2020 CE2437, including COPD without CRF group, and 14 May 2019 CE2288, including COPD with CRF), and all experimental procedures were performed according to the Declaration of Helsinki. All patients were admitted to our Institute for Pulmonary Rehabilitation, and data refer to the baseline evaluation. The CTRL GROUP subjects were recruited at the Department of Neuroscience, Biomedicine, and Movement of the University of Verona. The protocol (IRB#27111) was approved by the Institutional Review Board of the University of Verona. Written and informed consent was obtained from all participants.

Patients in the three groups of the study presented the following inclusion criteria:

COPD GROUP: patients had a diagnosis of COPD, according to the GOLD criteria (Goldcopd, 2024), with an FEV_1_/FVC ratio <70% and FEV_1_ < 80%, with a PaO_2_ ≥ 60 mmHg while breathing ambient air.

CRF GROUP: patients had: a) diagnosis of COPD as previously described, b) the prescription of 24 h/day of Long Term Oxygen Therapy (LTOT) for at least 3 months according to guideline recommendations (Hardinge et al., 2015; Forum Giovani Pneumologi, 2025); c) the diagnosis of CRF LTOT (PaO_2_ < 55–60 mm Hg) and/or chronic hypercapnia (PaCO_2_ > 45 mm Hg) in a stable clinical state, outside of acute exacerbations (Budweiser et al., 2008).

The O_2_ supply was delivered via nasal prongs from liquid oxygen from a base unit (a stationary system) during rest, and portable cylinders during daily activities. According to the leading Italian guidelines, O_2_ supply (L/min) was prescribed based on rest and exercise needs to achieve a SpO_2_ of greater than 91% (Forum Giovani Pneumologi, 2025).

CTRL GROUP: Subjects sex- and age-matched.

For all groups, exclusion criteria included the absence of any diagnosed major cardiac, respiratory, neurological, or orthopaedic pathology. Additionally, participants in the CRL group were required to be completely free of any form of respiratory disease.

### Procedures and measurements

**FIGURE 1:**
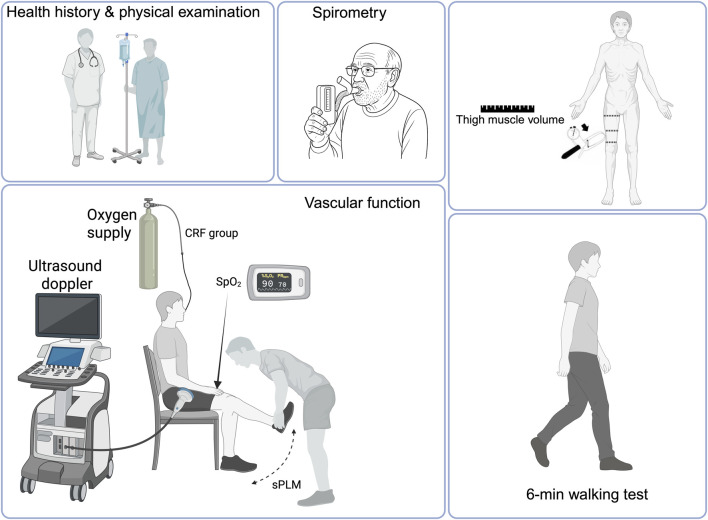
Schematic overview of the assessments carried out in the study for each subject included. Legend: **(A)** evaluation of inclusion and exclusion criteria and clinical stability; **(B)** Evaluation of lung function by spirometry; **(C)** Vascular function evaluation through an Eco Doppler scan during the single passive leg movement test (sPLM), on the right common femoral artery. It consisted of 30 s of resting baseline data recording, followed by one passive knee flexion and extension, lasting one second, after which the leg was maintained fully extended for 60 s. SpO_2_ and Heart Rate were recorded; **(D)** Leg muscle volume evaluation of the dominant thigh based on thigh circumferences (three sites: distal, middle, and proximal), thigh length, and skinfold measurements. **(E)** Physical function evaluation by the 6-min walking test.

The medical staff recruited COPD and CRF patients and performed a baseline screening, including health history, physical examination, and a skilled member of the team performed anthropometric assessment. During an initial familiarization session, all the selected subjects were accustomed to all study procedures. To determine the subject’s eligibility, one of the investigators moved the participants’ lower leg, beginning with ∼a 0° knee joint angle through a 90° range of motion. Subjects were excluded from further participation if they were not able to fully relax their leg during this passive manoeuvre. On a separate day, participants completed the entire assessment battery (Figure 1).

### Passive leg movement test

In detail, subjects rested in the upright-seated position for 20 min before the start of data collection and remained in this position throughout the vascular assessment test. Before starting and during the test procedure, oxyhaemoglobin saturation (SpO_2_) was estimated using pulse oximetry with a sensor (Check me lite, Gima, Milan, Italy). It is important to note that all the tests were performed in CRF subjects while breathing their usual O_2_ supplementation at rest, and the utilized inspiratory fraction of oxygen (FiO_2_) was recorded. FiO_2_ was estimated using the following formula: FiO_2_ (%) = 20 + (4 × O_2_ flow rate in L/min using nasal cannula) (Shapiro et al., 1982). Participants refrained from caffeine, alcohol, and exercise for 24 h before the experiment.

The vascular function was evaluated through the single Passive Leg Movement test (sPLM) test.

During the sPLM protocol, arterial blood velocity and vessel diameter were captured in the common right femoral artery, distal to the inguinal ligament and proximal to the deep and superficial femoral bifurcation, using a Doppler ultrasound system (Logiq V4, General Electric Medical Systems, Milwaukee, WI, US). The diameter of the common femoral artery was determined at a 90° angle along the central axis of the scanned area, measured as the distance (mm) between the intima-lumen interfaces for the anterior and posterior walls of the common femoral artery, as described in Gifford and Richardson (2017). The test consisted of a 30-s resting baseline echo Doppler recording, followed by one passive knee flexion and extension of the right leg, each lasting one second. Afterward, the leg was maintained in a fully extended position for 60 s (Venturelli et al., 2017). The total Doppler frame recording lasted 90 s, and the entire recording was included in the analysis. Blood velocity (Vmean) was measured using the linear probe, positioned to maintain an insolation angle of 60° or less, and the sample volume was cantered and maximized according to vessel size. LBF in millilitres per minute was calculated second-by-second as:

LBF = *V*mean ⋅ *π* ⋅ (vessel diameter/2)^2^ 60 for 30 s at rest and the 60 s following the sPLM.

The procedure was performed by an experienced healthcare professional involved in this area of research, who had received at least 6 months of training in carrying out the measurement. The test was repeated twice, with a rest interval of at least 20 min between each trial. The trial yielding the best result was considered.

Reliability of sPLM test: The latest evidence suggests that the sPLM test is a valid and efficient alternative to the traditional Passive Leg Movement (PLM), which typically involves repeated passive flexion and extension of the knee over a set period (e.g., 2 min), for screening peripheral vascular dysfunction, including in populations at risk for cardiovascular disease and diabetes. The sPLM test reliably detects attenuation in the hyperemic response related to age and disease, with diagnostic accuracy comparable to that of the continuous PLM protocol. Both in healthy and diseased populations, sPLM demonstrates clear discrimination between normal and impaired vascular function, with robust cut-off values for leg blood flow response that correlate strongly with those from the full PLM protocol (Broxterman et al., 2017; Hydren et al., 2019; Ives et al., 2020).

Data collection and analysis: Mean blood velocity (Vmean) was analysed with 1-Hz resolution on the Doppler ultrasound system (Logiq V4, General Electric Medical Systems, Milwaukee, WI, US) for 30 s at rest and the first 60 s following the initiation of sPLM. A 3-s rolling average was applied to the LBF data. Maximal absolute (peak), and delta peak (Δpeak) were determined for each subject in the vascular variables. Further details on the test are described elsewhere (Ives et al., 2020).

6-min walking test: Physical functioning capacity was evaluated by the 6-min walking test, conducted in a 30 m-long path following the procedure described by the ATS/ERS guidelines (Holland et al., 2014).

Thigh muscle volume: The volume of the dominant thigh was indirectly evaluated by the following formula: V = (L/12π) · (C12 + C22 + C32) − [(S − 0.4)/2] L · [(C1 + C2 + C3)/3 based on thigh circumferences (three sites: distal, middle, and proximal), thigh length, and skinfold measurements (Layec et al., 2014).

Spirometry: Lung function was evaluated by spirometry, including forced vital capacity (FVC), forced expiratory volume in the first second (FEV_1_), FEV_1_/FVC ratio, and residual volume (RV) (Holland et al., 2014) (Spirolab, Carefusion, Germany). Results were expressed as absolute and percentage of the predicted values according to Quanjer et al. (1993).

Arterial Blood Gas (ABG) (only in the COPD and CRF group): air ABG analysis was performed by obtaining a blood sample from the radial artery using a sterile syringe, whkle the patient was sitting and breathing room air. After the blood had been drawn, the sample was immediately expelled into a heparinized syringe, air bubbles were removed, and the syringe was sealed before being sent for analysis using a dedicated blood analyser (ABL90 series, Radiometer Medical APS, Denmark). Pressure was applied to the puncture site for at least 5 min to prevent bleeding and hematoma formation. The sample was analyzed for pH, partial pressures of oxygen (PaO_2_) and carbon dioxide (PaCO_2_), bicarbonate (HCO_3_^−^), and oxygen saturation.

### Statistical analysis

Statistical analysis was conducted using STATA 11 (StataCorp LLC, Texas 77845-4512, United States) and GraphPad Prism 8 version 8.4.3 for Windows (GraphPad Software, Boston, Massachusetts, United States).

Data were expressed as mean ± standard deviation (SD), except for categorical outcomes that were expressed as frequencies and percentages in each group. One-way ANOVA was used to describe significant differences among the three groups. In the presence of a significant ANOVA result, pairwise group comparisons were conducted using independent t-tests, with p-values adjusted using the Tukey correction to account for multiple comparisons.

Two-way ANOVA was used to analyse the hyperaemic response to sPLM in the three groups (interaction group x time). Correlations between baseline clinical data and the response to sPLM were performed by calculating the correlation matrix with the Pearson r coefficients. Statistical significance was set at p < 0.05.

## Results

### Subject characteristics

We included in our report 15 patients with moderate COPD (COPD GROUP), 15 patients with severe COPD at GOLD stage 4 with CRF (CRF GROUP), and a control group (CTRL GROUP) of 15 sex- and age-matched volunteers.

The baseline clinical characteristics of the patients and controls are shown in Table 1. Study participants were matched for sex (male 62.2%) and age. Comorbidities were equally distributed in the three groups, with more than half of the subjects having hypertension and being treated with antihypertensive drugs (58% of subjects). As expected, COPD and CRF patients had moderate to severe lung obstruction, which was significantly worse in comparison to COPD subjects, and showed a significant hypoxiemia when ABG was performed in room air. Control Healthy subjects had normal spirometry values. The majority of COPD patients were on triple inhaled bronchodilator therapy (73% of COPD, 93% of CRF). As expected, CRF had a much longer disease history, with more than twice as many years since diagnosis compared to COPD without CRF. CRF had been using oxygen supply for about 2 years, with an average flow rate of 3 L per minute.

**TABLE 1:** Subjects’ characteristics.

Variables	CRL	COPD	CRF	*p*
	N = 15	N = 15	N = 15	
Male, n (%)	10 (66.7%)	9 (60.0%)	9 (60.0%)	0.9154
Age (years)	70.7 ± 9.4	66.1 ± 8.8	67.7 ± 8.1	0.3456
BMI (Kg/m^2^)	24.6 ± 2.0	25.5 ± 5.3	23.2 ± 41	0.2966
Hypertension, n (%)	9 (60.0%)	8 (53.3%)	9 (60.0%)	0.9183
Dyslipidemic, n (%)	2 (13.3%)	1 (6.67%)	4 (26.7%)	0.3213
Diabetes, n (%)	1 (6.7%)	3 (20.0%)	2 (13.3%)	0.5526
CIRS 1 (score)	N/A	1.49 ± 0.19	1.77 ± 0.39	**0.0215**
CIRS 2 (score)	N/A	2.27 ± 0.97	3.00 ± 2.17	0.2461
Triple inhaled therapy, n (%)	0 (0%)	11 (73.3%)	14 (93.3%)	**<0.001**
LAMA + LABA inhaled therapy, n (%)	0 (0%)	4 (26.7%)	1 (6.67%)	**<0.001**
Oral antiplatelets drugs, n (%)	8 (53.3%)	7 (46.7%)	6 (40.0%)	0.7776
Antihypertensive drugs, n (%)	9 (60.0%)	8 (53.3%)	9 (60.0%)	0.9183
Never smoker, n (%)	8 (53.3%)	1 (6.7%)	1 (6.7%)	
Current smoker, n (%)	3 (20%)	13 (86.7%)	11 (73.3%)	**0.003**
Ex smoker, n (%)	4 (26.7%)	1 (6.7%)	3 (20%)	
Pack-years, n	N/A	39.6 ± 21.7	54.6 ± 25.4	0.0948
GOLD stage II, n (%)	0	8 (53.3%)	0	**<0.001**
GOLD stage III, n (%)	0	7 (46.7%)	0	**<0.001**
GOLD stage IV, n (%)	0	0	15 (100%)	**<0.001**
FEV_1_ (%pred)	87.9 ± 5.4	53.3 ± 11.4	30.6 ± 10.3	**<0.0001**
FVC (%pred)	90.7 ± 4.1	83.65 ± 15.61	69.4 ± 19.03	**0.0334**
FEV_1_/FVC (ratio)	77.7 ± 2.6	50.42 ± 8.88	35.60 ± 7.81	**<0.0001**
RV (%pred)	N/A	162.96 ± 47.48	192.65 ± 44.99	0.0897
pH	N/A	7.42 ± 0.03	7.42 ± 0.04	0.8474
PaO_2_ (mmHg)	N/A	72.62 ± 7.53	54.18 ± 4.30	**<0.0001**
PaCO_2_ (mmHg)	N/A	39.57 ± 3.59	45.71 ± 8.68	**0.0204**
Time from COPD diagnosis (years)	—	3.60 ± 2.26	10.53 ± 3.29	**<0.0001**
Time from starting oxygen (months)	—	—	25.33 ± 27.72	**<0.0001**
Day-time oxygen prescription (L/min)	0	0	3.03 ± 1.20	**<0.0001**
6MWD (meters)	554.3 ± 141.2	445.1 ± 115.3	277.7 ± 70.1	**<0.0001**
6MWD (%predicted)	116.3 ± 40.9	86.3 ± 16.4	54.1 ± 12.3	**<0.0001**

Data are expressed as mean ± sd or number (n) and percentage (%). CTRL, control; COPD, chronic obstructive pulmonary disease; CRF, chronic respiratory failure; BMI, body mass index; CIRS, Cumulative Illness Rating Score - 1 (Severity Index) and 2 (Comorbidity Complexity Index); LAMA, long-acting muscarinic antagonists; LABA, long-acting beta2 antagonists; Ex-smoker: ex-smoker for more than 3 months, na, not available; ICS, inhaled corticosteroids; FEV_1_, forced expiratory volume in the 1^st^ second; FVC, forced vital capacity; RV, residual volume, PaO_2_, arterial oxygen partial pressure; PaCO_2_, arterial carbon dioxide partial pressure; LTOT, long term oxygen therapy; 6MWD, 6-min walking distance; centimeters, L, liter; ml, milliliters; min, minutes; m, meter. N/A, not available. “*p*” refers to ANOVA or chi-square test. In bold are p with significant values.

### Vascular function

**FIGURE 2:**
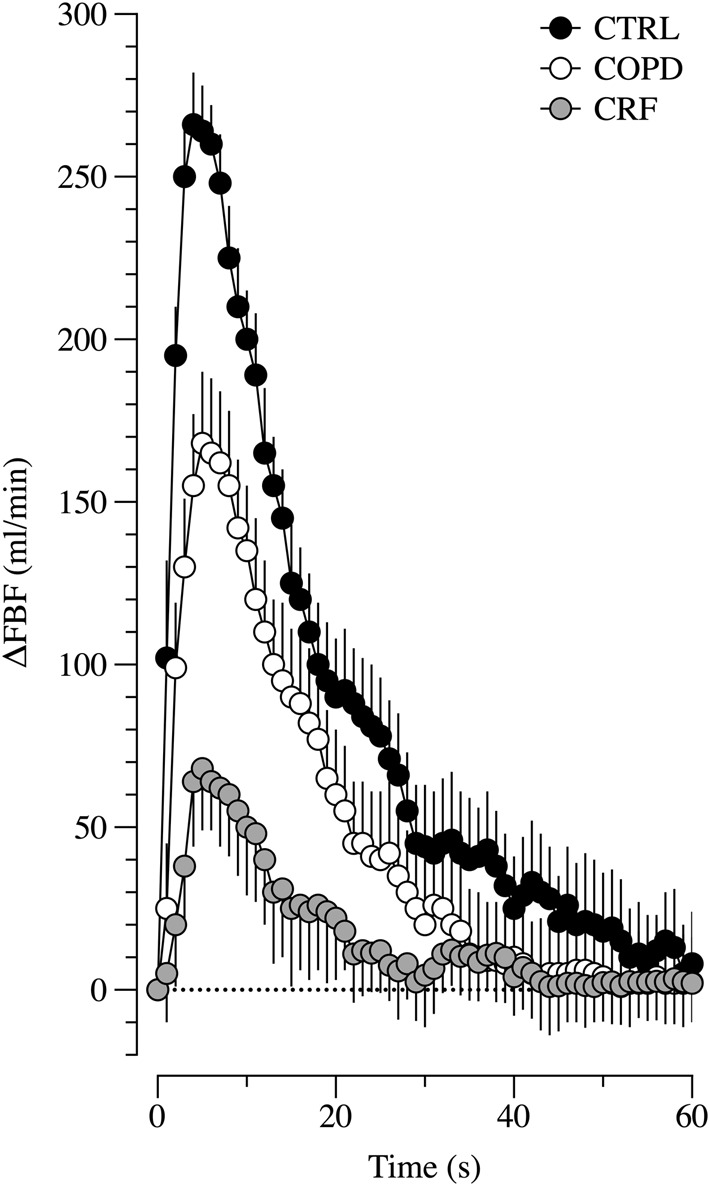
The change of femoral blood flow after sPLM manoeuvres among groups. Legend: ΔFBF, delta Femoral Blood Flow; CTRL, control group; COPD, COPD group; CRF, Chronic respiratory Failure group; s, seconds.

During sPLM, ΔLBF in the passively moved leg transiently increased from the 2nd to the 37th s in the CTRL and from the 4th to the 22nd s in the COPD. In the CRF subjects, the ΔLBF was significantly greater from rest from the 5th to the 19th second (Figure 2). The sPLM-induced hyperaemic response was significantly greater in the CTRL compared with the COPD between the 5th and the 14th s after the sPLM and between the 5th and the 24th s compared with the CRF (Figure 2). COPD exhibited a greater ΔLBF compared with the CRF from the 4th to the 22nd s. Table 2 shows the mean values of LBF at rest and following the sPLM in the three groups. Before starting the sPLM, in the resting state, the LBF was significantly reduced in the CRF compared to the COPD and CTRL, while no difference was observed between the COPD and CTRL (Table 2). The peak of LBF during the sPLM was significantly reduced in CRF compared to COPD and CTRL subjects (Table 2). Also, the COPD patients exhibited a significantly reduced sPLM-induced hyperemia (peak of LBF) compared to the CTRL. The values of ΔLBF follow the same trend as the peak LBF, exhibiting a progressive reduction of the sPLM-induced hyperemia from the CTRL to COPD and CRF.

**TABLE 2:** Vascular function expressed in absolute values, relative to leg muscle volume, and SpO_2_.

Variables		CTRL	COPD	CRF
Leg blood flow	Rest (mL/min)	252 ± 76	218 ± 145	164 ± 72*
	Peak (mL/min)	538 ± 159	436 ± 230§	234 ± 95*
	ΔPeak (mL/min)	268 ± 134	162 ± 115§	70 ± 38*
Leg blood flow/thigh volume	Rest (mL/min/cm^3^)	0.0312 ± 0.003	0.0321 ± 0.002	0.0287 ± 0.002
	Peak (mL/min/cm^3^)	0.0681 ± 0.009	0.0527 ± 0.008§	0.0403 ± 0.006*
	ΔPeak (mL/min/cm^3^)	0.0360 ± 0.005	0.0225 ± 0.008§	0.0116 ± 0.007*
Leg blood flow/SpO_2_	Rest (ml/min/%)	2.62 ± 0.65	2.29 ± 0.79	1.75 ± 0.45*
	Peak (ml/min/%)	5.59 ± 1.63	4.54 ± 2.38§	2.46 ± 0.99*
	ΔPeak (ml/min/%)	2.77 ± 1.35	1.69 ± 1.19§	0.73 ± 1.35*

Legend: Data are expressed as mean ± sd. CTRL, control; COPD, chronic obstructive pulmonary disease; CRF, chronic respiratory failure; *, significantly lower than COPD and CTRL; §, significantly lower than CTRL.

The peak LBF was moderately associated with FEV^1^ (r = 0.5432, p = 0.0001). There was also a moderate correlation with disease duration (r = 0.5062, p = 0.0004).

### Thigh muscle volume and vascular function

**FIGURE 3:**
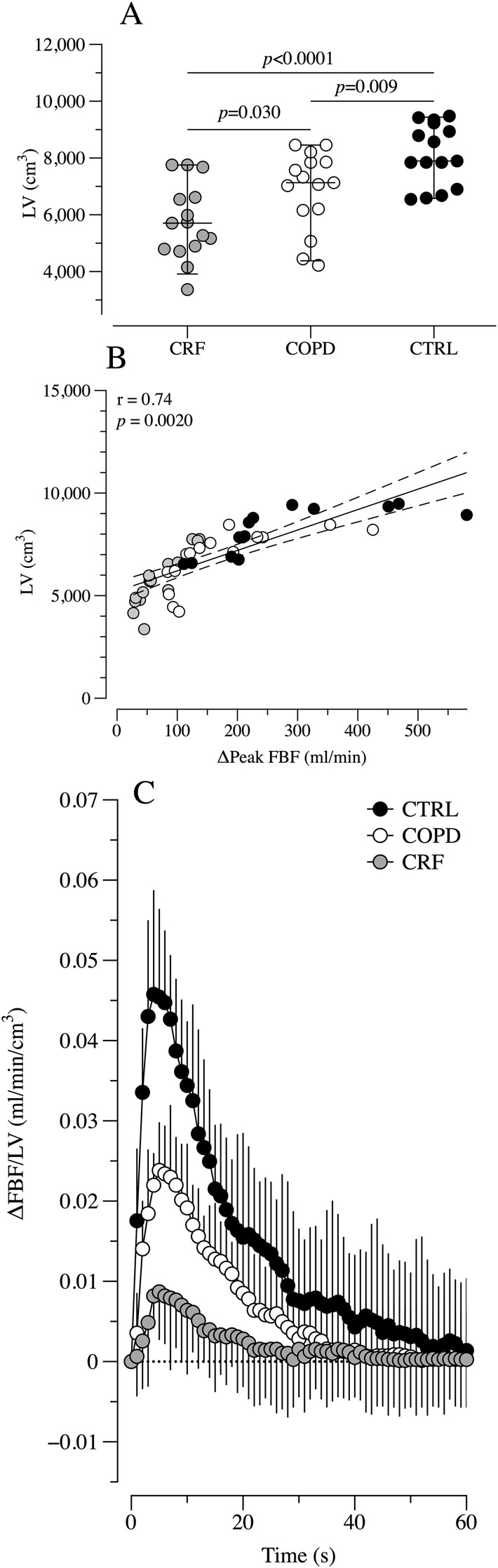
The thigh muscle volume **(A)**, its correlation with delta FBF **(B)** and ΔLBF after sPLM manoeuvres normalized by the LV **(C)** among groups. Legend: LV, Leg Volume; ΔFBF, delta Femoral Blood Flow; CTRL, control group; COPD, COPD group; CRF, Chronic respiratory Failure group; s, seconds.

The thigh muscle leg volume (LV) was significantly reduced in the CRF compared to COPD and CTRL (Figure 3, Panel A). Also, the COPD patients exhibited a reduced LV compared to the CTRL. Interestingly, LV and the ΔLBF following the sPLM were significantly correlated, r = 0.74, *p* = 0.0020 (Figure 3, Panel B). When the ΔLBF was normalized by the LV (Figure 3, Panel C), the differences in the sPLM-induced hyperemia remained significant between CTRL, COPD, and CRF (Table 2).

### Oxyhaemoglobin saturation and vascular function

As expected, the oxygen flow (FiO_2_) used during the test among groups was different, being higher in CRF (25.5% ± 0.02%) in comparison to COPD and CRL breathing in room air (FiO_2_ 21%), p < 0.001.

The oxygen saturation was different among groups (CRF: 95.1 ± 0.9, COPD: 96.1 ± 0.9, CRLT: 96.2 ± 1.1, p = 0.0044), CRF being significantly low in comparison to COPD (p = 0.014) and CRL (p = 0.009).

The hyperaemic response (ΔLBF) was positively associated with oxygen saturation (SpO_2_) (r = 0.4343, p = 0.0029), and inversely associated with oxygen supplementation prescribed (r = −0.5413, p < 0.0001).

When the ΔLBF was normalized by the SpO_2_, the differences in the sPLM-induced hyperemia remained significant between CTRL and the COPD and CRF groups (ANOVA p < 0.001) (Table 2).

### Exercise functional capacity and vascular function

As expected and in line with the literature exercise functional capacity was significantly worse in the CRF group, with fewer meters walked during the 6MWT (COPD vs. CRF, p = 0.0006; CTRL vs. COPD, p = 0.0296; CTRL vs. CRF; p < 0.0001; Table 1) and a lower percentage of distance walked compared to the expected value (COPD vs. CRF, p = 0.0051; CTRL vs. COPD, p = 0.0092; CTRL vs. CRF, p < 0.0001; Table 1). Interestingly, the sPLM hyperaemic response (ΔLBF) was found to be highly correlated to the exercise functional capacity assessed via the 6MWT (r = 0.70, *p* < 0.001).

### Smoking habits and vascular function

To investigate the potential influence of smoking habits on endothelial function, we conducted a correlation analysis between pack-years and sPLM hyperaemic response (ΔLBF) in the patient cohort (COPD + CRF). There was a moderate association, but it did not reach statistical significance (r = −0.31, p = 0.095).

## Discussion

In agreement with our hypothesis, subjects with CRF exhibited an attenuated sPLM-induced hyperemia compared to patients with less severe COPD and healthy controls. Interestingly, when the significantly attenuated ΔLBF was normalized to the thigh muscle volume, the difference in the hyperaemic response among the CRF, COPD, and CTRL groups was mitigated but not eliminated. Moreover, the significant reduction in SpO_2_ observed in COPD and CRF patients was positively correlated with the sPLM-induced hyperemia. However, even when ΔLBF was normalized to SpO_2_, the difference among the CRF, COPD, and CTRL groups was reduced but still present (Table 2). These data, therefore, provide evidence of an intrinsic vascular dysfunction during the progression of COPD, which does not completely depend on locomotor muscle wasting and the reduction of oxygen blood saturation typically observed in this clinical population.

### Evidence that the progression of pulmonary impairment is parallel to vascular dysfunction

The endothelium is an active and essential component of blood vessels, producing several key regulatory factors that are critical for maintaining systemic homeostasis (Förstermann, 2010). The mechanisms promoting endothelial dysfunction in the systemic and/or pulmonary circulation of COPD patients are diverse and include systemic inflammation, alterations in adhesion and pro-inflammatory molecules, oxidative stress, cellular senescence, and apoptosis. Nevertheless, the role of the endothelium in the onset and progression of COPD, and *vice versa*, is not yet fully understood (Screm et al., 2024; Abagnale et al., 2023; Siragusa et al., 2023). A systematic review and meta-analysis by Ambrosino et al. (2017) included eight studies evaluating brachial artery FMD in COPD patients and concluded that COPD severity was significantly and independently associated with endothelial dysfunction. However, since FMD is only partially mediated by NO availability, other vascular assessment tools such as PLM and sPLM (Venturelli et al., 2017; Rossman et al., 2016) may be more suitable for evaluating microvascular function in locomotor muscles, which are strongly affected by COPD and associated muscle disuse (Gea et al., 2013).

FMD measures conduit artery endothelial function, typically in the brachial artery, by quantifying vasodilation in response to increased shear stress following transient arterial occlusion. This response is largely nitric oxide (NO)-dependent and is considered a surrogate marker for cardiovascular risk.

sPLM instead assesses microvascular endothelial function in the lower limb by measuring the hyperemic response (increase in leg blood flow) following a single passive knee movement. This response is also predominantly NO-mediated, but reflects resistance artery function and microvascular health rather than conduit artery function.

The strength of sPLM is that it is a simple, NO-specific test for lower-limb microvascular function, distinct from FMD in physiological target and mechanism. Compared with FMD, sPLM is a simpler method that is less dependent on the operator and yields a single numerical value that is suitable for clinical screening (Broxterman et al., 2017; Hydren et al., 2019; Ives et al., 2020).

In this scenario, our findings describe a progressive impairment of sPLM-induced hyperemia in the legs of patients with CRF and COPD. In moderate COPD, our study showed that the ΔLBF following sPLM is consistent with the findings of Ives et al. (2020) who reported a ΔLBF during PLM of 142 ± 25 mL/min in patients with moderate COPD (mean FEV_1_ 53%) (Rossman et al., 2016). Our study extends these results, as it is the first to describe in detail the drastic reduction in vascular function in COPD patients with respiratory failure.

### Locomotor muscle sarcopenia and vascular function

Emerging literature describes a specific skeletal muscle dysfunction in patients with pulmonary disease (Gómez-Martínez et al., 2023). Sarcopenia, defined as the loss of muscle mass and function, frequently develops in the lower limbs of COPD patients and is closely associated with physical inactivity, chronic inflammation, hypoxemia, and nutritional deficiencies (Gómez-Martínez et al., 2023; Li et al., 2024). Increasing evidence suggests that vascular dysfunction may be both a contributor to and a consequence of muscle wasting in aging (de Lima et al., 2024). Moreover, as COPD progresses, reduced physical activity exacerbates muscle disuse, further diminishing local capillarization and altering microvascular function. Additionally, muscle atrophy itself may further impair vascular function, as skeletal muscle acts as a key endocrine organ, releasing myokines that influence endothelial health and local inflammation (Chen et al., 2025). Therefore, it is crucial to determine whether COPD-induced vascular dysfunction is driven primarily by skeletal muscle wasting or if an intrinsic vascular dysfunction also exists. In a previous study involving a different clinical population (individuals with spinal cord injury), we demonstrated that when the difference in blood flow response to PLM was normalized by muscle volume, vascular function appeared preserved (Venturelli et al., 2014). In contrast, the present investigation revealed that while the hyperaemic response to sPLM was correlated with thigh muscle volume in both patients and controls (Figure 3), normalizing by muscle volume did not eliminate the differences among the three groups. These results suggest that the recognized locomotor muscle loss in COPD patients is not the sole contributor to vascular dysfunction; rather, other intrinsic or systemic factors are likely involved in this physiological phenomenon.

### Chronic hypoxemia and vascular function

One of the key systemic characteristics of COPD development is chronic hypoxemia, characterized by sustained reduction in arterial oxygen levels (Vitacca et al., 2011). Chronic hypoxemia has profound effects on the autonomic nervous system, particularly through the activation and sensitization of peripheral chemoreceptors, which in turn activate the chemoreflex by regulating sympathetic outflow. A recent elegant study by Wan et al. (2022) demonstrated in healthy humans that acute hypoxia can modulate the hyperaemic response due to PLM, likely due to β-adrenergic activation that facilitates the peripheral haemodynamic response to PLM. Interestingly, in the COPD scenario, prolonged hypoxemia leads to chemoreflex sensitization, resulting in elevated sympathetic nerve activity (Wan et al., 2022). This increase in sympathetic tone contributes to peripheral vasoconstriction and, in turn, impaired endothelial function. Some studies have demonstrated that in aging and disuse, typical conditions exhibited in COPD patients, there is exaggerated muscle sympathetic nerve activity and blunted vasodilatory responses (Dinenno et al., 2005; Eickhoff et al., 2008; Spiesshoefer et al., 2022). It is important to note that chronic hypoxemia directly impairs vascular function by reducing NO bioavailability and promoting oxidative stress (Carpagnano et al., 2004). Crucially, the degree of chemoreflex sensitivity and vascular dysfunction appears to track with COPD severity. In early stages, compensatory mechanisms partially compensate vascular function, but in advanced stages (particularly in patients with CRF), vascular function is likely impacted by these physiological factors. Indeed, the results of the current study describe the close correlation between oxygen delivery (as indicated by oxyhemoglobin saturation during the test) and vascular dysfunction. Despite this fact, even after the normalization for the SpO_2_ during test, the difference among groups was still present, implying that other factors are likely contributing to this physiological phenomenon. For instance, only for the CRF group, the long-term oxygen therapy itself may have influenced the results, possibly acting as an additional contributor to endothelial dysfunction (Carpagnano et al., 2004). These factors, along with others we did not specifically look at (e.g., inflammatory status), may have contributed to the development of vascular dysfunction to varying degrees.

We should also note that the CRF group presented resting hypercapnia (PaCO_2_ = 45.7 mmHg). This is likely to affect central chemoreceptor activity, as hypercapnia is known to increase the sensitivity of central chemoreceptors to CO_2_, thereby enhancing ventilatory drive (Nattie and Li, 2012). Moreover, sustained hypercapnia may exert vascular effects, including endothelial dysfunction, impaired vasodilation, and increased sympathetic activation, which can contribute to altered peripheral vascular responses. The vascular dysfunction, therefore, appears to be a complex impairment, and, likely, several factors—including those highlighted here—act synergically (Najarian et al., 2000).

### Limitation

This study has several limitations. Firstly, its retrospective, cross-sectional, post-hoc design is a key constraint. As such, the study is largely descriptive and does not capture potential changes in the measured outcomes that may have occurred following the rehabilitative intervention. Secondly, although the sPLM test can assess peripheral vascular dysfunction, the absence of data on central hemodynamic responses prevents definitive conclusions being drawn about the contribution of multiple factors—such as autonomic regulation and chemoreflex activity—to the development of the observed vascular impairment. Future prospective studies incorporating complementary assessment methods are warranted to improve our understanding of this phenomenon. Thirdly, we only evaluated the muscle volume characteristics of the participants, without assessing the complex phenomenon of sarcopenia. A more detailed evaluation of muscle strength and performance could have revealed how different muscle impairments impact vascular function. Lastly, of the various factors that can impact vascular dysfunction (e.g., medication use, physical activity level, nutritional status, and hyperglycaemia), we were only able to conduct an in-depth evaluation of a subset. Regarding smoking habits, the available data did not allow for a detailed assessment, as pack-year data were unavailable for the control group. As a result, correlation analyses could only be performed in the patient population.

### Clinical and functional implications

Another novel and important observation of our study is the strong association we found between exercise intolerance (6-min walking test) and the LBF during the sPLM test (r = 0.703). This association highlights the potential role of endothelial function in facilitating oxygen delivery and promoting aerobic metabolism in peripheral muscles during exercise (Tschakovsky and Joyner, 2008). Taking into account that sPLM is predominantly mediated by NO (Venturelli et al., 2014), our results are very important because the role of this molecule for the exercise-induced hyperemia and metabolic regulation during physical activity is crucial. Studies have shown that NO contributes to forearm hyperemia during prolonged rhythmic hand gripping, with NO synthase blockade reducing blood flow by 20%–30% (Dyke et al., 1995). NO also regulates muscle glucose uptake during exercise independently of blood flow (Neunhäuserer et al., 2021).

In addition, our study confirms the close relationship between lung function and impaired vascular responsiveness, finding differences among groups with and without CRF and a strong correlation between the worsening of airway obstruction (FEV_1_) and the hyperemic response to the SPLM test. In this regard, previous studies, particularly those using the FMD technique, support our findings and emphasize the relationship between the severity of airflow obstruction and vascular dysfunction (Screm et al., 2024; Abagnale et al., 2023). Our research, using a different technique, outlines the progression of vascular dysfunction as the disease progresses. Other interesting findings include the strong association between NO-mediated hypoxemia and time since diagnosis, disease severity, and the amount of oxygen therapy prescribed. These findings may be valuable for designing effective cardiovascular prevention strategies in clinical and rehabilitation settings and may also stimulate further discussion about the role of vascular dysfunction in exercise intolerance in patients with very severe COPD. From a rehabilitative perspective, the sPLM test is highly useful as a rehabilitation tool due to the limited time it takes to perform and the fact that it directly assesses the lower limb district, which is usually trained during rehabilitation programmes. Additionally, due to its relative ease of execution and low learning curve, the sPLM test could be incorporated into the rehabilitation assessment battery. However, robust future studies on an adequately sized population are needed to determine the test’s sensitivity and define the minimum clinically significant difference that documents improvement.

In conclusion, our study supports the presence of a severe vascular dysfunction in COPD with CRF, which is only partly explained by locomotor muscle wasting and by hypoxemia observed in this clinical population.

## Data Availability

The raw data supporting the conclusions of this article will be made available by the authors, without undue reservation.
